# Generation of high-performance human cardiomyocytes and engineered heart tissues from extended pluripotent stem cells

**DOI:** 10.1038/s41421-022-00446-7

**Published:** 2022-10-11

**Authors:** Li Li, Zhongjun Wan, Ruxiang Wang, Yuxin Zhao, Yida Ye, Pengcheng Yang, Yan Qi, Wei Jiang, Lin Cai, Donghui Zhang

**Affiliations:** 1 State Key Laboratory of Biocatalysis and Enzyme Engineering, National & Local Joint Engineering Research Center of High-throughput Drug Screening Technology, Hubei Province Key Laboratory of Biotechnology of Chinese Traditional Medicine, School of Life Science, Hubei University, Wuhan, Hubei China; 2 Department of Biological Repositories, Frontier Science Center for Immunology and Metabolism, Medical Research Institute, Zhongnan Hospital of Wuhan University, Wuhan University, Wuhan, Hubei China

**Keywords:** Induced pluripotent stem cells, Stem-cell differentiation

Dear Editor,

The availability of functional human cardiomyocytes is essential for cardiac disease modeling, drug screening, and cell therapy, whereas donor human cardiomyocytes are incredibly scarce. Human embryonic stem cells (ESCs) and induced pluripotent stem cells (iPSCs) are widely used to provide an unlimited supply of cardiomyocytes through differentiation schemes, such as modulating Wnt/β-catenin signaling^1,2^. However, the use of ESCs/iPSCs face some challenges, such as heterogeneity, the poor survival rate, and differentiation bias, limiting their application^3,4^. To solve these problems, massive efforts have been made, including optimizing culture conditions and deriving more powerful new pluripotent cell types^3^. Mouse and human extended pluripotent stem cells (EPSC) established by Dr. Deng’s group^5^ in 2017 have the bidirectional chimeric ability that contributes to both embryonic and extraembryonic lineages. Furthermore, human EPSCs have been shown superior chimeric ability in monkey embryos very recently^6^, which further demonstrates their outstanding developmental potential. In addition, EPSC-derived hepatocytes showed improved function and a more similar transcriptome to human primary hepatocytes than ESC/iPSC-derived hepatocytes^7^. However, whether EPSCs can efficiently generate other lineages such as cardiomyocytes and how the EPSC-derived cardiomyocytes (EPSC-CMs) function compared with ESC/iPSC-CMs have not been studied yet.

Recently, we have established a Matrigel-based feeder-free method to convert and maintain human EPSCs^8^, which would largely facilitate the application. In this study, we successfully generated functional cardiomyocytes and engineered heart tissue from human EPSCs (experimental design shown in Fig. 1a). In addition, we examined the mitochondrial function, calcium handling, and contractility properties of EPSC-CMs at monolayer and microtissue levels compared with ESC/iPSC-CM controls. We further evaluated the performance of EPSC-CMs in the myocardial infarction model in nude rats.

**Fig. 1:**
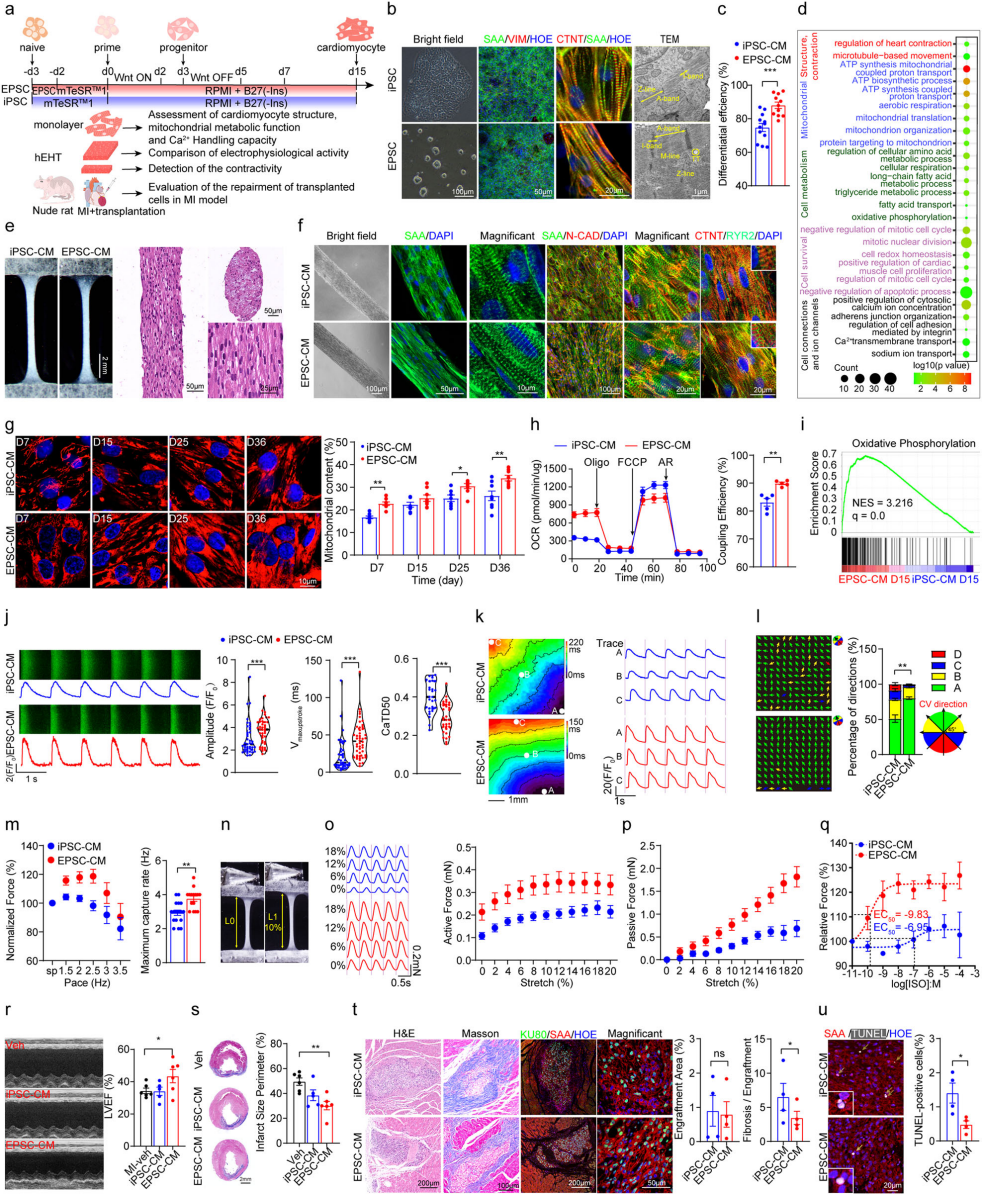
High-performance of human cardiomyocytes and EHTs generated by human EPSCs. **a** Scheme of the research design and procedures. For cardiomyocyte differentiation, EPSCs were pretreated with mTeSR^TM^1 medium for 2 days and then administrated by Wnt signaling modulators. ESCs/iPSCs were cultured in mTeSR^TM^1 for 3 days, followed by the same differentiation protocol. On day 15, the generated cardiomyocytes were digested and reseeded on a monolayer or engineered heart tissue for functional assessment. On days 20–22, the cardiomyocytes were injected into the infarcted nude rat heart for injury recovery experiments. **b** Bright field, immunofluorescence staining of cardiomyocyte markers SAA and CTNT, fibroblast marker Vimentin on day 15 (middle), and transmission electron microscopy showing myofibrillar alignment in EHTs of both cell types. **c** Statistics of CTNT+ rate at day 15 of differentiation by flow cytometric analysis (*n* = 9 for iPSC group, *n* = 11 for EPSC group). **d** Gene ontology analysis of cardiac-related biological processes for up-regulated genes in EPSC-CMs vs iPSC-CMs on day 15 of differentiation. (*n* = 3 biological replicates in each group). **e** Representative light microscopy images of cardiac bundles from iPSC-CMs and EPSC-CMs; H&E staining images from longitudinal and cross-sections showing well-arranged myofiber. **f** Bright field and immunofluorescence staining of SAA and N-cadherin showing tight conjunction among cardiomyocytes in EPSC-CM- and iPSC-CM-derived bundles; while well-arranged RYR2 only presented in a 2-week EPSC-CM-constructed bundle. **g** Representative confocal images and quantitative analysis (right) of live MitoTracker staining in reseeded iPSC-CMs and EPSC-CMs with the indicated time points. *n* = 6–8. **h** Real-time oxygen consumption rate (OCR) measurements of iPSC-CMs and EPSC-CMs reseeded for 8 days (left), and calculation of coupling efficiency (right) by Seahorse extracellular flux analyzer (*n* = 5 per group). **i** GSEA showing enrichment plots of gene expression signatures of oxidative phosphorylation pathway in EPSC-CMs vs iPSC-CMs on day 15. **j** Representative images and Ca^2+^ transients (left) of iPSC-CMs and EPSC-CMs. Cells were loaded with Fluo-4 AM and paced for 15 s at 1 Hz one time, and then Ca^2+^ transients were recorded post-pacing by confocal line scan imaging. Quantification of Ca^2+^ transient amplitude of fluorescence changes, maximum upstroke speed, and duration at 50% repolarization (CaTD50) (right) in both cell types. More than 50 cells from three biological replicates per group were recorded. **k**, **l** Optical mapping of 2-week iPSC-CM and EPSC-CM patches stimulated at 1 Hz pacing. Isochronal activation maps (**k** left) and calcium transient traces (**k** right), as well as vector illustrations and statistics of distributions towards propagating directions (**l**) were shown, respectively. *n* = 9 for iPSC-CM group, *n* = 11 for EPSC-CM group. **m** Active force traces in iPSC-CM- and EPSC-CM-derived bundles spontaneously or at various pacing rates (**m** left) and maximum captured pacing rate (**m** right). *n* = 9–10 in each group. **n** Representative images of the cardiac bundle at original state or 10% stretch. **o**, **p** Corresponding active force (**o**) and passive force (**p**) in 2-week cardiac bundle made with iPSC-CMs and EPSC-CMs during the progressive stretch of an electrical stimulation (2 Hz), respectively. **q** Contractile force amplitude in response to different isoproterenol concentration (*n* = 5). **r** Representative echocardiographic images (left) and quantification of left ventricular ejection fraction (LVEF) (right) at 6 weeks after LAD coronary artery ligation and iPSC-CM/EPSC-CM grafting. *n* = 6 rats in MI + vehicle group, *n* = 5 rats in MI + iPSC-CM group, *n* = 6 rats in MI + EPSC-CM group. **s** Representative images (left) and quantification (right) of the infarcted area from Masson staining from heart sections in each group. **t** Representative histological images and quantifications of iPSC-CM and EPSC-CM’s engraftments in the hearts at 6 weeks after cell transplantation. H&E, Masson and immunostaining images of KU80 and SAA showed the survived engraftments in rat hearts. The EPSC-CM group exhibited comparable engraftment size but a reduced percentage of fibrotic area in grafts (*n* = 4 rats per group). **u** Representative images and quantification (right) of TUNEL staining in the grafts (*n* = 4 rats per group).

As reported^5,7^, EPSCs grew as smaller colonies and expressed pluripotent markers OCT4 and SSEA4 (Supplementary Fig. S1a). To generate EPSC-induced cardiomyocytes, we first tried but failed to get beating cardiomyocytes with standard protocols^1,9^. Then, by converting EPSC culture into mTeSR^TM^1 medium (a commercial feeder-free maintenance medium for human ESCs/iPSCs) for two days (named as EPMC cells) before initial differentiation (preconditioning), we successfully achieved EPSC-derived cardiomyocytes (Fig. 1b; Supplementary Fig. S1b and Video S1). EPSCs showed stable and repeatable cardiomyocyte production (Fig. 1c), with high efficiency determined by flow cytometric analysis for mesodermal marker BRACHYURY (Supplementary Fig. S1c), cardiac progenitor markers NKX2.5 and ISL1 (Supplementary Fig. S1d). Of note, EPSCs generated a higher percentage of CTNT^+^ cardiomyocytes (Fig. 1c; Supplementary Fig. S1e) in a robust manner (Supplementary Figs. S1f, S4b). Simultaneously, EPSCs could generate massive beating cardiomyocytes with initial cell confluency ranging from 70%–100%. In contrast, iPSCs could only generate more than half of the beating cells within the optimal range of 70%–85% confluence (Supplementary Fig. S1f). Moreover, we found that treatment with FGF2 and TGFβ instead of mTeSR^TM^1 for preconditioning also significantly increased the CM generation efficiency to about 85%, indicating FGF2 and TGFβ favored cardiomyocyte differentiation from EPSCs (Supplementary Fig. S1g, h and Video S2). In addition, EPSH9, another EPSC line converted from ESC line H9, could also be differentiated into CTNT^+^ cardiomyocytes by regulating Wnt signaling (Supplementary Fig. S4a and Video S3), confirming the advantage of EPSCs as seeding cells for cardiomyocyte differentiation.

To determine why EPSCs could be differentiated into cardiomyocytes with higher efficiency, we compared the transcriptome of EPSCs, EPMCs, and iPSCs (Supplementary Fig. S2a, b). Firstly, we found that EPMC has distinct naïve and primed stemness gene expression clusters, which separates from EPSC and iPSC (Supplementary Fig. S2c, d), indicating three types of cells with different stemness characteristics. More importantly, gene set enrichment analysis (GSEA) showed that the top 15 enriched terms in the EPMCs group included organ development and cell fate commitment. Dr. Deng’s previous research proposed that EPSC transformed stage 1 cells were similar to day 6 to day 8 implantation epiblast, whereas iPSCs/ESCs showed identical to day 10 to day 12 epiblast cells^7^. This founding suggested the EPSC-derived EPMC has enhanced pluripotency potential than iPSC (Supplementary Fig. S2e), which indicated EPMCs were in a state more prone to differentiate, consistent with a previous report about hepatic differentiation from EPSCs^7^. We demonstrated that EPSCs could effectively differentiate into cardiomyocytes with essential preconditioning. Then the raising questions became whether the EPSC-CM has higher maturity than iPSC-CM.

Next, we compared the structure and mitochondrial function of EPSC-CMs and iPSC-CMs at monolayer and microtissue levels. The gene ontology (GO) of transcriptome analysis showed that EPSC-CMs exhibited enhanced expression of gene related to sarcomere organization and metabolic maturation compared with iPSC-CMs after 15 days of differentiation (Fig. 1d; Supplementary Fig. S3a–c). In addition, although no significant difference in sarcomere structure was observed between EPSC-CMs and iPSC-CMs, the EPSC-CM-derived engineered heart tissue (EHT) (Fig. 1e) showed a more mature cellular structure than iPSC-CM-derived EHT in terms of the polar distribution of N-Cadherin, RYR2 marked T-tubule, and significantly increased mitochondrial mass (Fig. 1f, g; Supplementary Fig. S3d). Furthermore, this relatively abundant mitochondria content presented a higher baseline coupling efficiency assessed by a Seahorse Bioscience XF Analyzer (Fig. 1h; Supplementary Fig. S3e) and the oxidative phosphorylation enrichment determined by GSEA (Fig. 1i). Also, we observed similar results with more abundant mitochondria in EPSH9-CMs than in the parental H9-CMs (Supplementary Fig. S4c). Together, these data indicated that EPSC-CMs exhibited more mature morphology and improved function as compared with ESC/iPSC-CMs.

To test whether the EPSC-CMs present a more functional physiological phenotype than iPSC-CMs, we compared the calcium handling ability in both cultured CMs and EHTs. The results showed that EPSC-CMs exhibited increased calcium transient amplitude and shorter Ca^2+^ decay time, indicating a relatively rapid and mature calcium cycling (Fig. 1j). Similar results also appeared in H9-CM and EPSH9-CM comparisons (Supplementary Fig. S4d). Next, optical mapping evaluated the tissue-level electro-conduction properties in iPSC-CM- and EPSC-CM-derived EHTs. Though conduction velocity was comparable, EPSC-CM EHTs displayed better uniformity of propagating direction(Fig. 1k, l), higher action potential amplitude, and shortened rising time at 1 Hz pacing and additional isoproterenol stimuli (Supplementary Fig. S5a–d). Finally, we further assessed the contractility properties of iPSC-CM- and EPSC-CM-derived bundle EHTs. After two weeks of cultivation, EPSC-CM and iPSC-CM EHT groups showed comparable widths (Supplementary Fig. S5e, f and Video S4), while EPSC bundles had a higher contraction amplitude and reduced peak-to-peak time under spontaneous conditions and ISO stimulation (Supplementary Figs. S5g–j). In addition, we used a force transducer to define the contractility properties in iPSC-CM and EPSC-CM EHTs directly. The EPSC-derived bundles showed positive pacing responses, reached a 1.2-fold force increase at 2.5 Hz on average, and showed a higher maximum capture rate (Fig. 1m). We also evaluated inotropic response during progressive stretching (Fig. 1n) and isoproterenol stimulation and found EPSC-CM EHTs showed a more pronounced positive and negative inotropic response in progressive stretching (Fig. 1o, p). Furthermore, the EPSC-CM EHTs presented an incredibly enhanced IC50 as 1000× sensitivity in ISO response (Fig. 1q). Our results indicated that EHTs derived from EPSC-CMs exhibited structural and functional maturation compared to iPSC-CM controls.

At last, to investigate whether EPSC-CMs could survive in the myocardium and potentially improve ischemia heart injury, we used the myocardial infarction (MI) nude rat model with intramuscular injection of EPSC-CMs or iPSC-CMs. First, after injecting EPSC-CMs (Supplementary Fig. S6a) into normal nude rat hearts for 4 weeks, we confirmed the survival of engraftments in rat hearts by H&E staining (Supplementary Fig. S6b) and immunostaining of human KU80 and SAA (Supplementary Fig. S6c). Next, MI followed by iPSC-CMs or EPSC-CMs transplantation or vehicle transplantation was performed in two other batches of nude rats. We performed echocardiography before MI and two days after cell transplantation, followed by histological analysis. Our results showed that the three groups had comparable LVEF both before and 2 days after MI, indicating the stability of the surgical procedure (Supplementary Fig. S6d and Table S1). Two days after cell transplantation, we observed no significant differences in graft size, fibrosis in engraftment, and apoptosis between iPSC-CM and EPSC-CM groups, indicating consistency in cell injection (Supplementary Fig. S6e, f). To further evaluate the long-term effects of EPSC-CM transplantation, we conducted MI plus cell injection for another batch of nude rats. After 6 weeks they were subjected to echocardiography and further histological analysis. Compared with the MI-vehicle group, the left ventricular ejection fraction (LVEF) and left ventricular fraction shortening (LVFS) in the EPSC-CM group were significantly improved at 6 weeks post-injection (Fig. 1r; Supplementary Table S2), altogether with the decrease in infarcted size (Fig. 1s), which implicated that transplantation of EPSC-CMs could help alleviate cardiac injury in MI model. We further found that while the two cell types formed comparable sizes of graft implants (Fig. 1t) and possessed similar mitochondrial contents in the engraftments (Supplementary Fig. S6g), EPSC-derived grafts showed less fibrotic replacement (Fig. 1t) and lower apoptosis rate (Fig. 1u). Thus, our results indicate that transplantation of EPSC-derived cardiomyocytes might be better than iPSC-CM controls in the long-term injury recovery.

Our study provides a new and promising source of human cardiomyocytes through newly discovered stem cells, namely EPSCs. Furthermore, cardiomyocytes derived from EPSCs showed high efficiency and robustness and exhibited improved mitochondrial function, calcium handling, and contractility properties at monolayer and microtissue levels in contrast to ESC/iPSC-CMs. Most importantly, EPSC-CMs restored cardiac function and performed better than iPSC-CMs in the nude rat MI model, thus being a better candidate cell source for regenerative therapy. The reason why EPSC-derived cardiomyocytes perform better than iPSC-CM is probably due to the difference in initial status between iPSC and EPSC, which could further affect developmental potential. This work will contribute to a wider understanding of expectation for human induced cardiomyocyte clinical study in future.

## Supplementary information

Supplementary information

Supplementary Video S1

Supplementary Video S2

Supplementary Video S3

Supplementary Video S4
