# Supplementary material for: Generation of high-performance human cardiomyocytes and engineered heart tissues from extended pluripotent stem cells
Source: Cell Discov. 2022 Oct 11;8:105. doi: 10.1038/s41421-022-00446-7 (PMC9553887; doi:10.1038/s41421-022-00446-7)
Supplement: Supplementary file 1 — Supplementary information [file 41421_2022_446_MOESM1_ESM.pdf]

Supplementary Information

Supplementary Figure S1

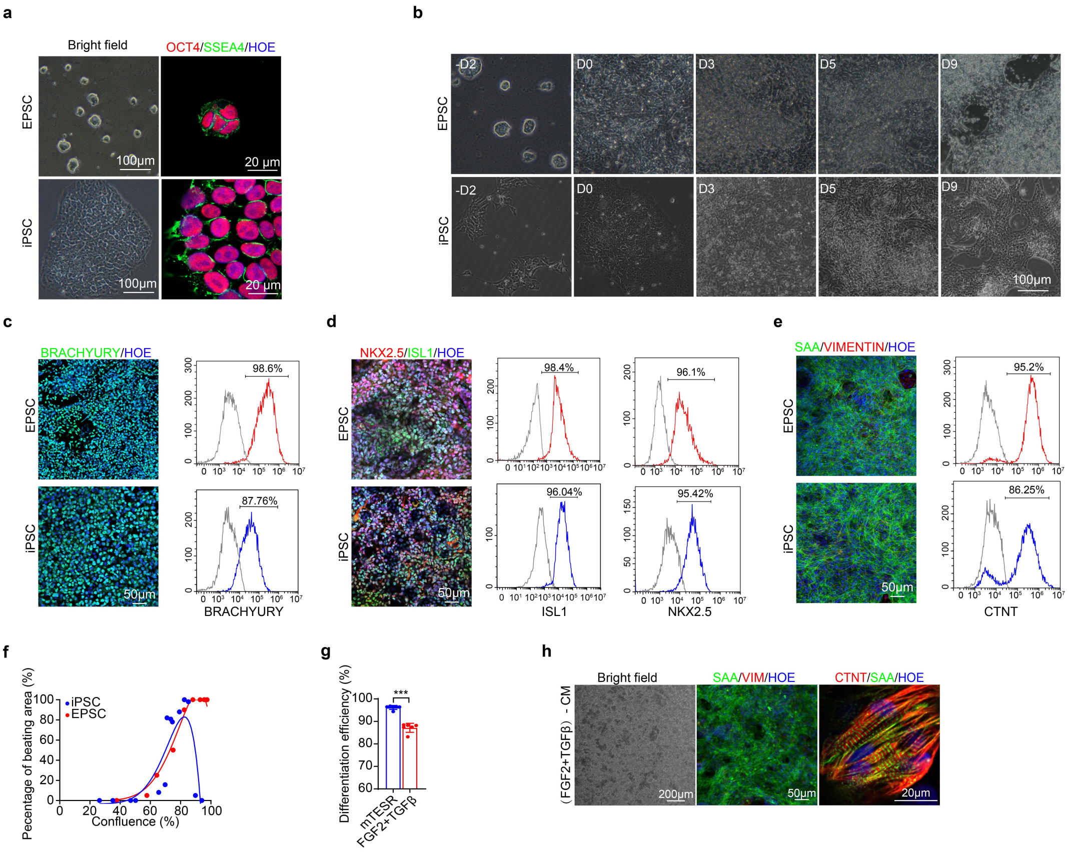

Supplementary Fig. S1. Stepwise differentiation of human EPSC/iPSC into cardiomyocyte.

a Bright field, immunofluorescence staining of pluripotency markers OCT4 (red) and SSEA4 (green). **b** Representative phase contrast images showing changes of cell morphology on day -2, 0, 3, 5, 9 (beating cardiomyocytes) under same differentiation protocol in EPSC and iPSC groups. Scale bar, 100 μm. **c-e** Immunofluorescence staining of mesoderm markers BRACHYURY (green) on day 2 (c), cardiac progenitor cell marker NKX2.5 (red) and ISL1 (green) on day 9 (d), and cardiomyocyte marker SAA (green) and fibroblast marker VIMENTIN (red) on day 14 (e). The cell nucleus was marked by Hoechst (Blue). The positive rate of BRACHYURY, NKX2.5, ISL1, CTNT in EPSC and iPSC groups was respectively determined by flow cytometric analysis. **f** Statistics of cardiomyocytes beating areas on day 15 at different cell confluences for differentiation initiation in EPSC and iPSC groups. **g** Statistics of CTNT positive rate at day 15 of EPSC differentiation by flow cytometry which transformed through mTeSR^{TM}1 or RPMI 1640 + B27(+ins) + FGF2 + TGFβ medium for 48 hours (n = 6). **h** Representative bright field and Immunofluorescence staining of cardiomyocyte marker SAA (green) and fibroblast marker VIMENTIN (red) on day 15, SAA (green) and CTNT (red) of reseeding monolayer cells on day 5 by transformed through 1640 + B27(+ins) with FGF2 + TGFβ medium for 48 hours.

Supplementary Figure S2

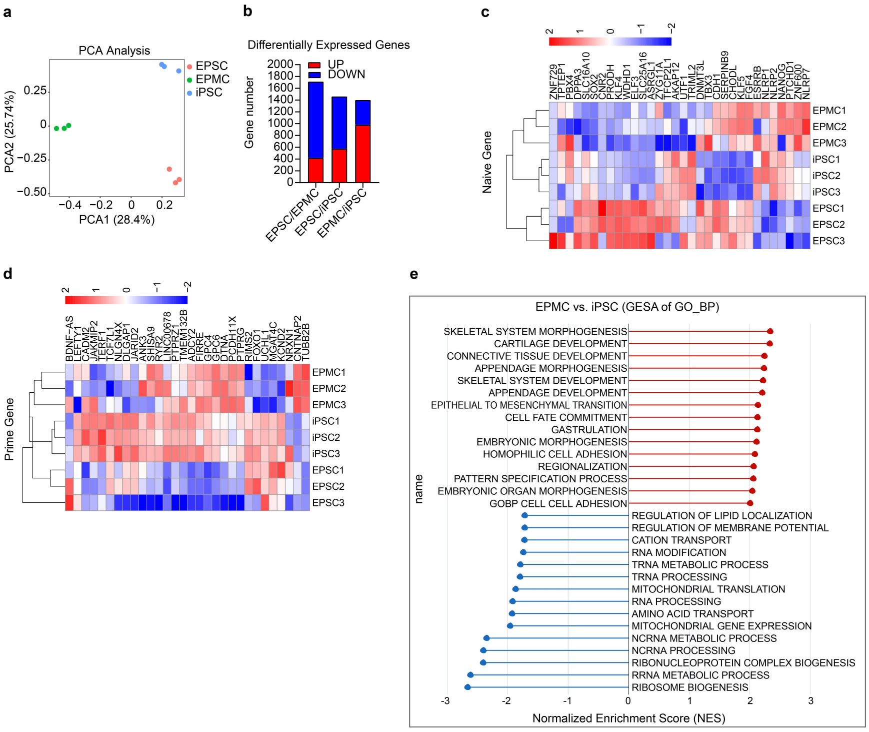

Supplementary Fig. S2. Different differentiation potentials among EPSC, iPSC and EPMC.

a PCA plot of RNA-seq data from EPSC, iPSC and EPMC (pre-conditioned EPSC in mTeSR^{TM}1 medium for 2 days) groups. **b** Statistics of significantly differentially expressed genes number (cutoff: $\log_2$(fold-change) ≥ 1 and *P*-value < 0.05). **c-d** Heatmaps showing the expression of naïve genes and primed genes from modules C and D (Yang, Y. et al. *Cell*. 2017) in three groups. **e** GSEA showing the top 15 significant signatures. Red bars indicated the biological process (BP) enriched in the EPMC group and blue bars indicated those enriched in the iPSC group. RNA-seq experiments were performed on n = 3 batches.

Supplementary Figure S3

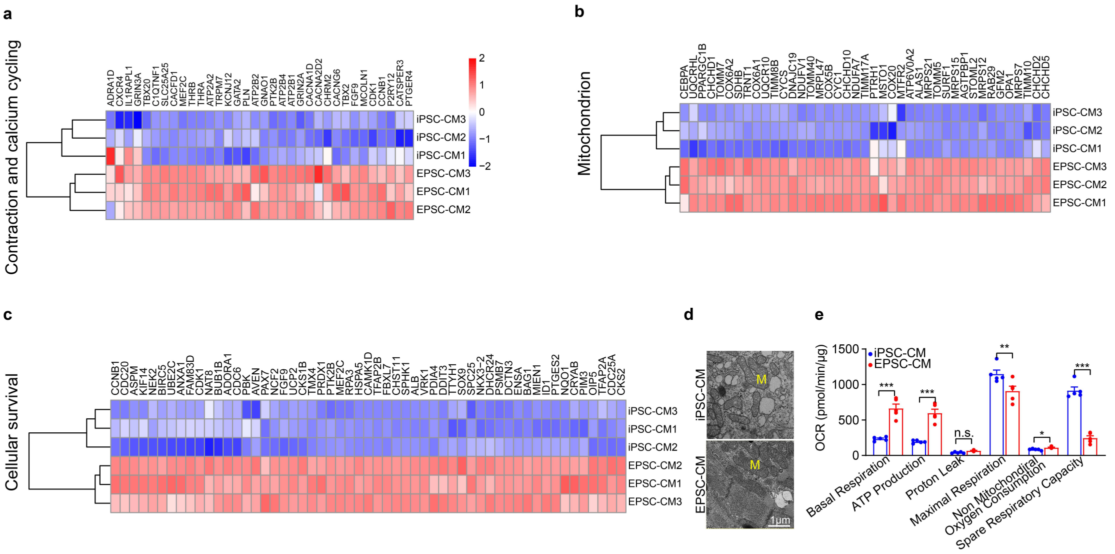

Supplementary Fig. S3. Comparison of Gene expression and mitochondrial function on 15-day EPSC-CMs and iPSC-CMs.

a-c Heatmaps of significantly higher expressed genes involved in distinct features of CM contraction and calcium cycling (a), mitochondrial synthesis and metabolism (b) and cellular survival (c) in EPSC-CM group contrast to iPSC-CM group. RNA-seq experiment was performed on n = 3 batches. **d** Transmission electron microscopy showing differently abundant and condensed mitochondria in two types of EHTs. **e** Real-time oxygen consumption rate (OCR) measurements of iPSC-CMs and EPSC-CMs reseeded for 8 days, and calculated basal respiration, ATP production, proton leak, maximal respiration, non-mitochondrial oxygen consumption, and spare respiratory capacity by Seahorse extracellular flux analyzer (n = 5 per group).

Supplementary Figure S4

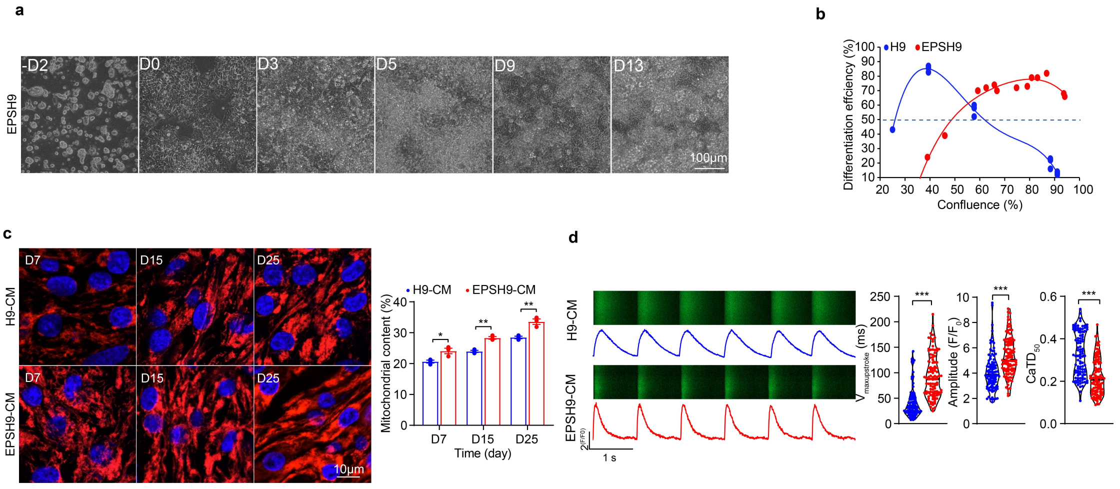

Supplementary Fig. S4. Comparison between human H9 ESC line and its derived EPSH9 in cardiomyocyte differentiation efficiency and derived CM function.

a Representative image showing that EPSH9 cell line could differentiate into beating cardiomyocyte on day 13 by the same protocol as EPSC (WTC line). Scale bar, 100 μm. **b** Statistics of CTNT positive rate on day 15 at different cell confluences for differentiation initiation by flow cytometry in H9 and EPSH9 groups. **c** Representative confocal images and quantitative analysis (right) of live MitoTracker staining in reseeding monolayer H9-CMs and EPSH9-CMs with the indicated time points. n = 10 - 12 images from 3 independent batches. **d** Representative images and Ca^{2+} transients (left) of H9-CMs and EPSH9-CMs. Cells were loaded with Fluo-4 AM and paced for 15 s at 1 Hz one time. Quantification of Ca^{2+} transient amplitude of fluorescence changes, maximum upstroke speed, and duration at 50% repolarization (CaTD50) (right) in two groups. More than 100 cells from three biological replicates per group were recorded.

Supplementary Figure S5

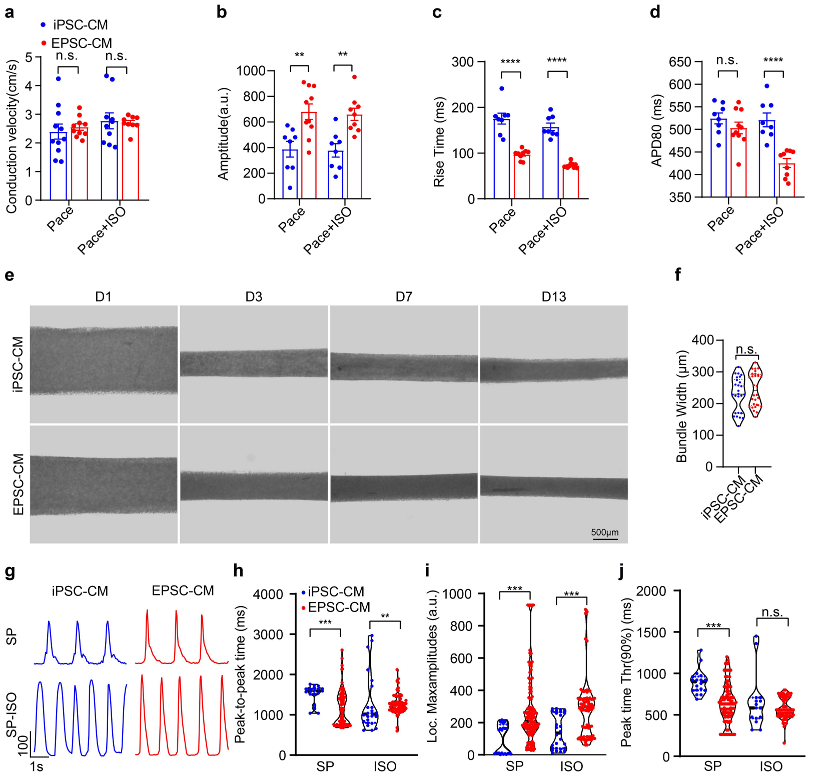

Supplementary Fig. S5. Comparison of electrical and contractive properties between EPSC- and iPSC-derived EHTs.

a-d 2-week iPSC and EPSC EHT patches stimulated with 1Hz electrically and Isoproterenol recorded by optical mapping. Quantitative analysis of conduction velocity (a) (n = 11 in iPSC-CM group, n = 10 in EPSC-CM group), amplitude (b), rise time (c) and APD80 (d) (n = 8 in iPSC-CM group, n = 10 in EPSC-CM group). **e** Representative light microscopy images of EPSC-CM/iPSC-CM EHT bundles on days 1, 3, 7, and 13. **f** Quantitative analysis of bundle widths in both cell types before further functional experiments (n = 26 in iPSC-CM group, n = 30 in EPSC-CM group). **g-j** Analysis of iPSC and EPSC EHT bundles 10s beating video recording spontaneously or induced by Isoproterenol. Representative waveforms (g) and quantitative analysis of peak-to-peak time (h), maximum amplitude (i), and the peak time of 90% amplitude (j). (n = 5 in iPSC-CM group, n = 12 in EPSC-CM group).

Supplementary Figure S6

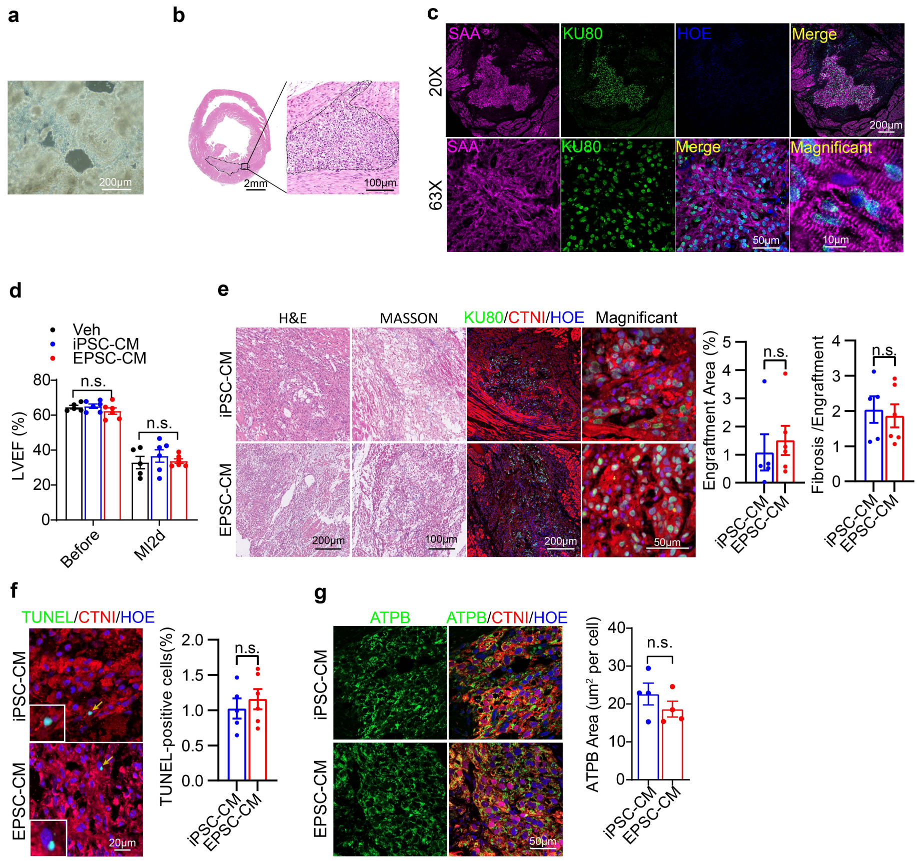

Supplementary Fig. S6. EPSC-derived cardiomyocytes survived in nude rat hearts after cell transplantation.

a Representative light microscopy image of EPSC-derived cardiomyocyte clumps before transplantation. **b** H&E staining images showing that EPSC-derived cardiomyocytes engrafted in non-surgical rat hearts after four weeks of transplantation. **c** Representative immunostaining images of SAA and KU80 showing that EPSC-derived cardiomyocytes survived in nude rat hearts. **d** Quantification of left ventricular ejection fraction (LVEF) assessed by echocardiography before MI and 2 days after MI plus cell transplantation, respectively. n = 5 rats in MI + vehicle group, n = 6 rats in MI + iPSC-CM group, n = 6 rats in MI+ EPSC-CM group. **e** Representative histological images and quantifications of iPSC-CM and EPSC-CM's engraftments in the hearts at 2 days after cell transplantation. H&E, Masson and immunostaining images of KU80 and CTNI showing viable grafts in rat hearts. The EPSC-CM group exhibited comparable engraftment size and fibrosis in grafts (n = 5 rats in MI + iPSC-CM group, n = 6 rats in MI+ EPSC-CM group). **f** Representative images (left) and quantification (right) of TUNEL staining in the grafts (n = 5 rats in MI + iPSC-CM group, n = 6 rats in MI+ EPSC-CM group). **g** Representative ATPB and CTNI immunostaining images and quantification analysis indicating mitochondrial content in the engrafted cardiomyocyte of both iPSC-CM and EPSC-CM groups 6 weeks after cell transplantation. The cell nucleus was marked by Hoechst (Blue). (n = 4 rats per group).

Supplementary Table S1. Echocardiographic data prior to and 2 days after MI and cell transplantation

	Baseline			MI2d		
	Veh	iPSC-CM	EPSC-CM	Veh	iPSC-CM	EPSC-CM
N	5	6	6	5	6	6
HR (bmp)	351.40±11.40	381.67±9.48	379.83±13.10	425.40±19.84	421.67±10.65	431.17±11.82
LVEDV (ml)	0.56±0.03	0.48±0.02[#]	0.56±0.07	0.59±0.07	0.69±0.12	0.51±0.04
LVESV (ml)	0.36±0.02	0.31±0.02	0.35±0.04	0.20±0.04	0.27±0.07	0.17±0.02
LVEF (%)	64.61±1.00	65.15±1.25	62.37±1.82	32.89±3.53	36.66±3.59	33.77±1.31
LVFS (%)	30.82±0.71	31.15±0.87	29.37±1.21	13.38±1.64	15.23±1.73	13.65±0.62
IVSd (mm)	0.87±0.06	0.96±0.05	0.92±0.04	0.76±0.07	0.87±0.08	1.01±0.06[*]
LVIDd (mm)	6.22±0.10	5.88±0.08[#]	6.19±0.28	6.32±0.25	6.62±0.41	5.98±0.17
LVPWd (mm)	1.05±0.08	1.24±0.04[#]	1.07±0.04	1.09±0.07	1.15±0.05	1.11±0.07
IVSs (mm)	1.38±0.07	1.55±0.06	1.46±0.03	0.83±0.07	1.10±0.11	1.16±0.06
LVIDs (mm)	4.30±0.05	4.04±0.04[##]	4.38±0.21	5.47±0.18	5.58±0.26	5.16±0.13
LVPWs (mm)	1.51±0.04	1.57±0.07	1.51±0.10	1.26±0.05	1.32±0.02	1.43±0.09

Data are presented as mean ± SEM. N: Number; HR: Heart Rate; LVEDV: Left Ventricular End Diastolic Volume; LVESV: Left Ventricular End Systolic Volume; LVEF: Left Ventricular Ejection Fraction; LVFS: Left Ventricular Fractional Shortening; IVSd: Interventricular Septal Thickness at Diastole; LVIDd: Left Ventricular end-diastolic Internal Dimension; LVPWd: Left Ventricular Posterior Wall end-diastole; IVSs: Interventricular Septal Thickness at Systole; LVIDs: Left Ventricular end-systolic Internal Dimension; EDV: End-Diastolic Volume; LVPWs: Left Ventricular Posterior Wall end-systole.

* $p < 0.05$, EPSC-CM vs Veh; # $p < 0.05$,## $p < 0.01$, iPSC-CM vs Veh.

Supplementary Table S2. Echocardiographic data of 6 weeks after MI and cell transplantation

	Veh	iPSC-CM	EPSC-CM
N	6	5	6
HR (bmp)	344.50±12.70	372.80±20.11	408.67±17.59
LVEDV (ml)	1.37±0.09	1.30±0.12	1.39±0.20
LVESV (ml)	0.90±0.07	0.86±0.09	0.79±0.13
EF (%)	34.29±1.69	34.08±2.57	43.44±4.11*
FS (%)	14.22±0.82	14.07±1.24	18.93±2.34*
IVSd (mm)	0.68±0.09	0.66±0.07	0.75±0.12
LVIDd (mm)	8.14±0.21	8.40±0.29	8.55±0.47
LVPWd (mm)	1.06±0.09	0.86±0.02	1.06±0.07
IVSs (mm)	0.81±0.12	0.71±0.06	0.91±0.17
LVIDs (mm)	7.36±0.20	7.22±0.28	6.93±0.44
LVPWs (mm)	1.37±0.09	1.21±0.12	1.36±0.07

Data are presented as mean ± SEM. N: Number; HR: Heart Rate; LVEDV: Left Ventricular End Diastolic Volume; LVESV: Left Ventricular End Systolic Volume; LVEF: Left Ventricular Ejection Fraction; LVFS: Left Ventricular Fractional Shortening; IVSd: Interventricular Septal Thickness at Diastole; LVIDd: Left Ventricular end-diastolic Internal Dimension; LVPWd: Left Ventricular Posterior Wall end-diastole; IVSs: Interventricular Septal Thickness at Systole; LVIDs: Left Ventricular end-systolic Internal Dimension; EDV: End-Diastolic Volume; LVPWs: Left Ventricular Posterior Wall end-systole.

* $p < 0.05$, EPSC-CM vs Veh.

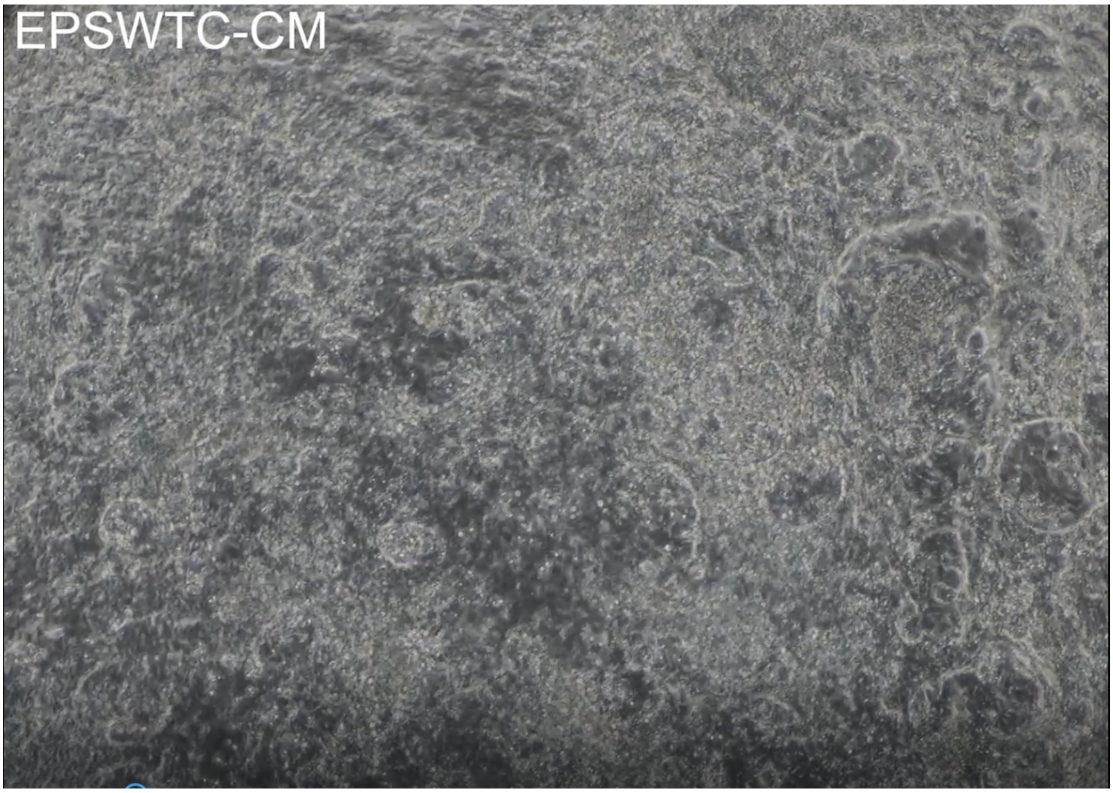

Supplementary Video S1. EPSC-CM on day 15. EPSCs (derived from WTC cell line) were pretreated with mTeSR1™ for 2 d before initial differentiation, then exposure to 2.5-7.5 μM CHIR 99021 at day 0 (for 48h) and 5 μM IWR-1 + 2.5 μM IWP-2 at day 3 (for 48h). Video was recorded on day 15.

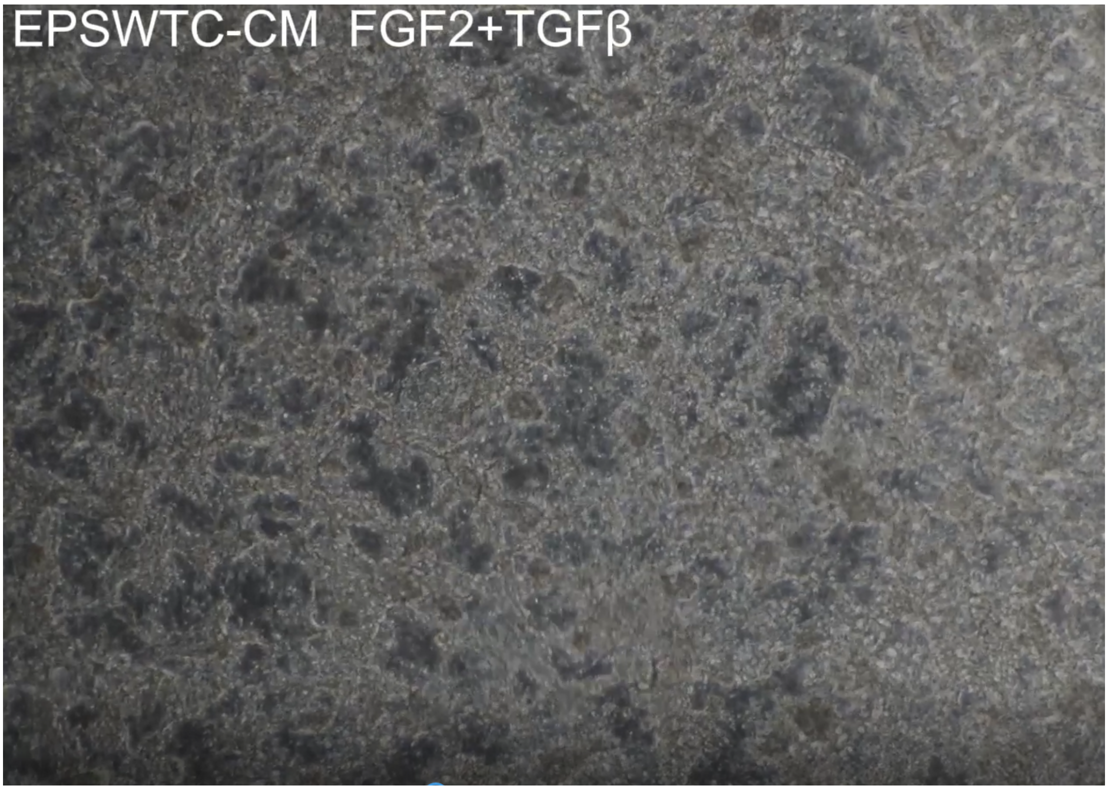

Supplementary Video S2. Day 15 cardiomyocyte derived from EPSC pre-treated with FGF2 and TGFβ. EPSCs (derived from WTC cell line) were cultured in RPMI 1640 medium and B27(+ins) supplimented with (100 ng/mL) FGF2 and (2 μg/mL)TGFβ for 2 days before initial differentiation, then exposure to 2.5-7.5 μM CHIR 99021 at day 0 (for 48h) and 5 μM IWR-1 + 2.5 μM IWP-2 at day 3 (for 48h). Video was recorded on day 15.

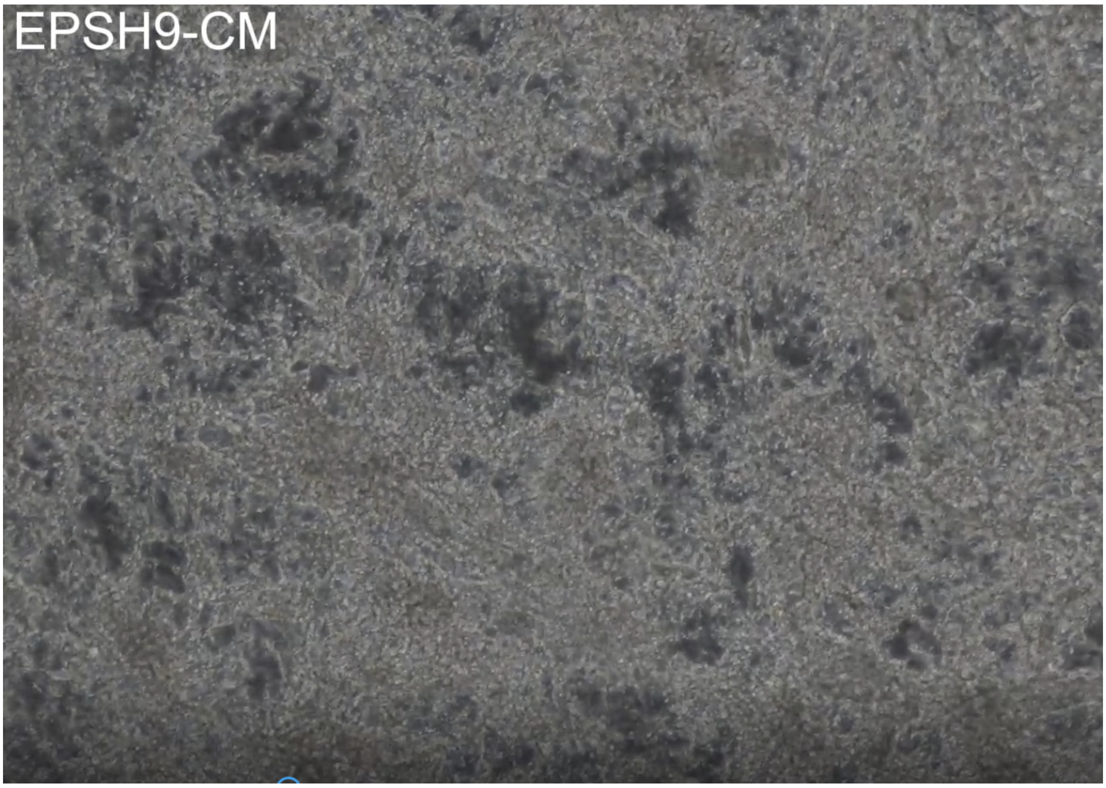

Supplementary Video S3. EPSH9-CM on day 15. EPSH9 (derived from H9 cell line) were pretreated with mTeSR^{TM}1 for 2 days before initial differentiation, then exposure to 2.5-7.5 μM CHIR 99021 at day 0 (for 48h) and 5 μM IWR-1 + 2.5 μM IWP-2 at day 3 (for 48h). Video was recorded on day 15.

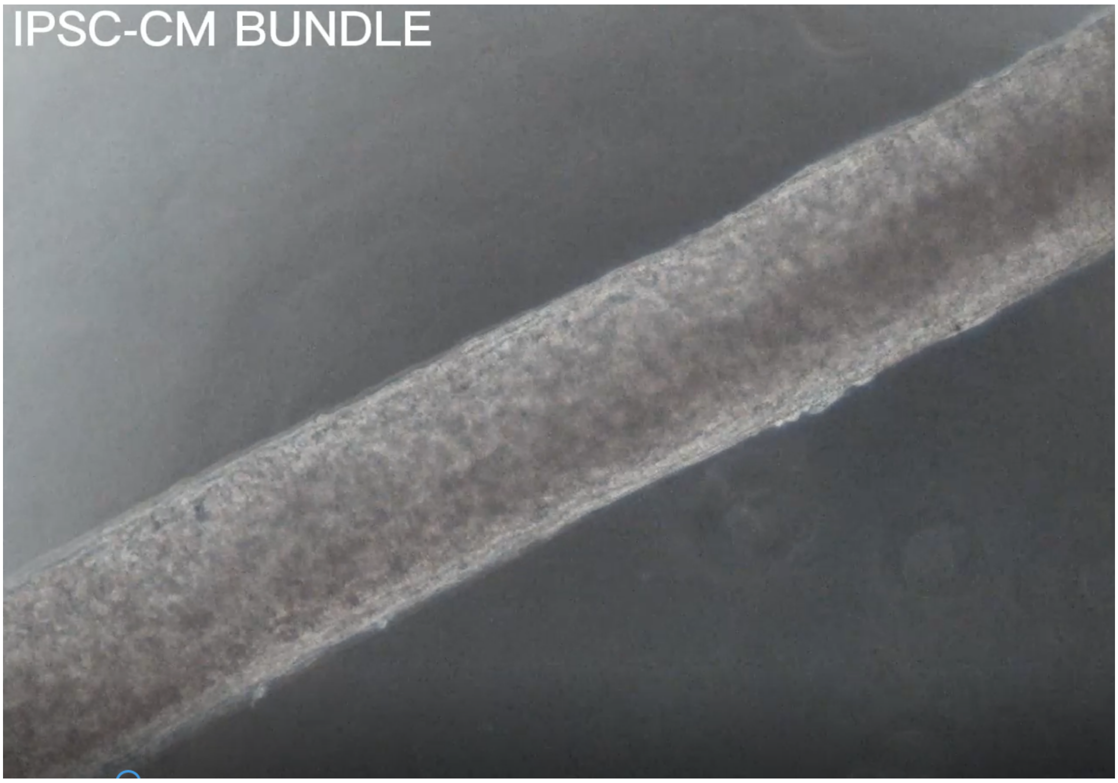

Supplementary Video S4. iPSC-CM- and EPSC-CM-derived EHTs on day 14. CMs were constructed to EHT bundles with hydrogel and cultured for 2 weeks before video recording.

Materials and Methods

Maintenance and conversion of hiPSC and hESC to hEPSC

Human iPSC line WTC (Gladstone Institute, from fibroblast), and human ESC line H9 (gifted from Medical Research Institute of Wuhan University) were used. iPSC and ESC cells were converted into EPSC in feeder-free conditions as previously described[1]. In brief, Cells were dissociated to single cells with Versene (Thermo Fisher, 15040066), and seeded on matrigel (1:30, corning, 354262) -coated plates. Cells were cultured in mTeSR™1(Stemcell, 85850) medium for the first 24 h and then changed into human EPSC culture medium. A total of 50 mL of human EPSC medium was prepared as below: 22.5 mL Neurobasal™ Medium (Gibco, 21103049), 22.5 mL KnockOut™ DMEM/F-12 (Gibco, 12660012), 10 μL (S)-(+)-Dimethindene maleate (10 nM, Tocris, 1425), 5 μL IWR-1 (10 mM, Tocris, 3532), 5 μL CHIR-99021 (10 mM, Tocris, 4423), 10 μL Y-27632 (10 mM, Stem cell, 72308), 10 μL Minocycline hydrochloride (10 mM, Tocris, 3268), 25 μL Human LIF (20 ug/mL, Peprotech, 300-05), 250 μL N2 (BasalMedia, S430J4), 500 μL B27(-VA) (BasalMedia, S441J7), 500 μL GLUTAMAX-1 (Gibco, 35050061), 500 μL NEAA (Gibco, 11140050), 500 μL Penicillin Streptomycin (Gibco, 15140122), 2.5 mL KSR (Gibco, A3181501) and 100 μL β-ME (Gibco, 21985023). Passaging at a high density (1:3 ratio) was preferred for the first three passages and the conversion was completed in about 12 generations. Then human EPSC could be propagated well in human EPSC medium.

Human EPSC culture and cardiomyocyte differentiation

Human EPSCs were seeded on Matrigel-coated (1:30, corning, 354262) plates and cultured in serum-free EPSC medium under 5% CO_2 at 37°C. The medium was changed every day, and washed once with DPBS (Gibco, C14190500BT) before changing. Human EPSC were passaged by TrypLE™ Express Enzyme (Gibco, 12604021) every 3-4 days.

For cardiomyocyte differentiation, human EPSC were digested into single cells with TrypLE™ Express Enzyme and seeded on Matrigel-coated (1:100) wells at a density of $3*10^4$ cells/cm^2 using human EPSC medium for 24 hours. Then the cells were treated with mTeSR™1 medium (Stemcell, 85850) for 48 hours, or cultured in RPMI 1640 medium (Gibco, C11875500BT) combined B27(+ins) (BasalMedia, S441J7) with 100 ng/mL FGF2 (Peprotech, 100-18B) and 2μg/mL TGFβ (PeproTech, 100-21C) for 2 days before initial differentiation. When the confluency of the culture reached 50% to 90%, cells were treated with 2.5-7.5 μM CHIR 99021 (Tocris, 4423) in differentiation medium for 24-48 hours. 50 mL differentiation basal medium was prepared as follows: 48.5 mL Basic RPMI 1640 Medium (Gibco, C11875500BT) with 1% Penicillin Streptomycin, 1 mL B27-insulin (BasalMedia, X064E4) and 200 μL L-Ascorbic acid 2-phosphate sesquimagnesium salt hydrate (50mg/mL, sigma, A8960). After 48 h, CHIR99021 was removed for another 24 hours. Next, cells were treated with 5 μM IWR-1 (Tocris, 3532) and 2.5 μM IWP-2 (Tocris, 3533) in the differentiation medium for 48 hours. Then, IWR-1 and IWP-2 were removed, and the differentiation medium was changed every two days from day 5.

Flow cytometry

hEPSC-derived cardiomyocytes were digested into single cells with TrypLE™ Express Enzyme at room temperature for 5 minutes. Harvested cells were washed twice with DPBS

in a 15 mL centrifuge tube and then fixed with 4% paraformaldehyde (Biosharp, BL539A) for 30 minutes. After washing for three times with DPBS, the cells were then blocked with blocking buffer (DPBS containing 5% donkey serum and 0.2% Triton X-100) at 4°C overnight. Cells were incubated with PE-CTNT (1:20, BD Pharmingen, 564767) in a blocking buffer at 4°C for 1 hour. After washing twice with DPBS, the cells were resuspended with 50-120 μL DPBS for flow cytometry analysis.

Construction and culture of three-dimensional CM-derived EHTs

To generate 3D human cardiac tissue patches, 9×9 mm^2 polydimethylsiloxane (PDMS, Dow Corning) was microfabricated as previously described[2]. Hydrogel solution (24 μL fibrinogen (10 mg/mL), 12 μL Matrigel, 24 μL 2× cardiac media) was mixed with 1.5×10^6 cells in 58 μL 1× cardiac media and 2.4 μL thrombin to obtain a total of 120 μL of cell/gel solution. Cell/gel solution was added to PDMS molds containing a Velcro frame, then incubated at 37°C for 30 minutes to polymerize. The resulting cardiac patches were cultured with rocking in early cardiac medium (1:1 with medium 1 and medium 2; Medium 1: RPMI 1640 with 1 mM sodium pyruvate (Gibco, 11360070), 2 mg/mL 6-Aminocaproic acid (Sigma, A2504), 0.1 mM non-essential amino acids (Gibco, 11140050), 200 μg/ml Ascorbic Acid (Sigma A8960), and 0.45 mM 1-Thioglycerol (Sigma, M6145); Medium 2: no glucose DMEM with 4 μM lactate) for 5 days. 10 μM Y-27632 (Stem cell, 72308) and 5% FBS was added on day 0 of patch culture to increase viability of the iPSC-CMs. Y-27632 and FBS was removed after 24 hours of culture. On the sixth day, cardiac patches were cultured with rocking in later cardiac medium (DMEM, low glucose, GlutaMAX™ Supplement, pyruvate with 2 mg/mL 6-Aminocaproic acid (Sigma, A2504), 0.1 mM non-essential amino acids (Gibco, 11140050), 200 μg/ml Ascorbic Acid (Sigma A8960), 5% FBS and 0.45 mM 1-Thioglycerol (Sigma, M6145)) and media was changed every 2 days.To generate bundle EHTs, 11.5×8 mm^2 polydimethylsiloxane (PDMS, Dow Corning) molds with Velcro frame (1.2 mm high) were used. Using the hydrogel system described above, each bundle contained 45 μL hydrogel mixture and 0.5×10^6 cells. The same culture system was applied to bundles, and relevant functions were assessed after 14 days of cultivation.

Seahorse XF24 metabolic flux analysis

The proper cell density was determined by a preliminary experiment. Then 1×10^5 cells were seeded on a Cell Culture Microplates (Agilent) and treated with Calibrate Sensor Cartridges with Calibrant for 8 days, followed by twice washing using OCR buffer solution. The culture plate was placed under the Calibrate Sensor Cartridges and OCR detection reagent (2 μM oligomycin, 2 μM FCCP, 0.5 μM rotenone + antimyocin A) was added to Calibrate Sensor Cartridges. The values were measured in Seahorse XFe24 (Agilent). At last, the cells were lysed with RIPA for BCA quantification to normalize the total cell numbers.

Mitochondrial morphology analysis

Cardiomyocytes seeded on a confocal dish were incubated with 1μM MitoTracker® Red CMXRos (ThermoFisher Scientific, M7512) in the dark for 10 minutes. After washing in an indicator-free medium to remove the excess dye, the cells were subsequently stained

with Hoechst for 15 minutes and washed for 3 times. After that, cells were treated with the original medium containing 2.5 μM (-)-Blebbistatin (Sigma, B0560) to inhibit contractions for 5 minutes in the incubator. ZEISS LSM 980 inverted laser scanning confocal microscope with a 63×-oil objective was used for confocal fluorescence imaging. The images were analyzed using the Fiji-ImageJ software.

Ca^{2+} transient measurement

CMs were incubated with 2.5μM Fluo-4, AM (ThermoFisher Scientific, F14201) for 30 minutes. After incubating, CMs were washed in indicator-free medium to remove the excessive dye for 3 times, and then treated with the original medium containing 2.5 μM (-)-Blebbistatin (Sigma, B0560) to inhibit contractions for 5 min. CMs were electrically stimulated at 1 Hz to produce steady-state conditions. ZEISS LSM 980 inverted laser scanning confocal microscope with a 63X-oil objective was used for confocal fluorescence imaging by line scan. To evaluate isoproterenol response, ISO was loaded for a final concentration of 1×10^{-6} M.

Contraction force measurement, force frequency, stretch and adrenergic response of EHTs

A customized force measurement apparatus was used to measure contractile force during field stimulation. Briefly, to assess the force-frequency and force-length relationship, bundles were immersed in 37 °C Tyrode's solution with 1.8 mM $CaCl_2$ and stimulated by the electrical pulse of 10 V and different frequencies (0, 0.5Hz, 1Hz, 1.5Hz, 2Hz, 2.5Hz, 3Hz, 3.5Hz, 4Hz). Then the bundle was stretched by linear actuator from 0% to 20% above the resting culture length in 2% increments. Inotropic responsiveness of the bundles was tested in the presence of β-adrenergic agonist isoproterenol ranging from 1×10^{-11} to 1×10^{-4} M in 1.8 mM Ca^{2+} Tyrode's solution during 2Hz electrical stimulation at 4% stretch. Contractile force data were analyzed using a custom MATLAB program.

Force assessment of EHT-bundle by video analysis

Spontaneous beating of EHT-bundles was recorded in the microscope (4×) for 10 seconds. Then 1×10^{-6} M ISO was loading for further data recording. The results were then analyzed by MYOCYTER plugin, to assess the amplitude, peak time threshold (90%, 50%, 20%), and contraction frequency of the bundle.

Immunofluorescence staining

For immunofluorescence staining experiments, cells seeded on confocal dishes and EHTs were cultured for 7 and 15 days, respectively. The medium was removed and washed once or twice with DPBS. Cells or EHTs were fixed with 4% paraformaldehyde (Biosharp, BL539A) and incubated at room temperature for 15 minutes. After washing with DPBS for three times for 5 minutes, the cells or EHTs were then blocked with blocking buffer (DPBS containing 5% donkey serum and 0.2% Triton X-100) at 4°C overnight. Cells were incubated with of blocking buffer containing primary antibody at 4°C overnight. After washing twice with DPBS, cells were incubated at room temperature for 2 hours (in the shade) with a blocking buffer containing the secondary antibody. After washing 3 times with DPBS, the nucleus was then stained with DAPI or Hoechst at room temperature for about

10 minutes, followed washing three times with DPBS. Immunofluorescence staining were then observed by ZEISS LSM 980 and image data were analyzed using Fiji-ImageJ software.

Optical mapping

Optical mapping of transmembrane potentials was performed after 15 days of culture using our established methods[3]. Cardiac patches were incubated with Rhod-2 AM (Thermo Fisher, R1245MP, 2.5 μM) at 37°C for 25 minutes. After washing twice with DPBS, patches were treated with the original medium containing 2.5 μM Blebbistatin (Sigma, B0560) to inhibit cardiomyocyte contraction and eliminate motion artifacts during recording. 10-second episodes of electrical activity induced by stimulation with point electrode were recorded in microscopic (16×) mode using a 594-channel photodiode array. Conduction velocity (CV) data analysis was performed using BV Ana software and APD data analysis was performed using OMapScope software.

Transmission electron microscopy

The EHT-bundle cultured for 20 days was directly fixed with a sufficient amount of pre-cooling 2.5% glutaraldehyde for 1 hour (cells were completely immersed in glutaraldehyde). Following complete natural sedimentation for 1 hour, samples were incubated in 1mL new pre-cooled 2.5% glutaraldehyde after supernatant was absorbed and stored in the refrigerator at 4°C. This process should be gentle and quick, to make sure not to damage cells. Then samples were sent to the Electron Microscopy and Histology Facility of Shanghai DanDa company for subsequent preparation and imaging.

RNA-seq and gene expression analysis

RNA-seq was conducted by using Npvaseq PE150 (Jike, Beijing, China) with Paired-End reads based on Illumina Novaseq. In brief, total RNA was extracted from 1) EPSC/iPSC-derived cardiomyocytes on day15; 2) EPSC, EPMC, and iPSC before initiation of cardiomyocyte differentiation, using TRIzol (ThermoFisher Scientific, 15596026) according to manufacturer’s instruction and RNA sequencing was conducted. Each group had 3 biological replicates. Raw RNA-seq data will be publicly available on https://ngdc.cncb.ac.cn/ (Project number: HRA001708). Cutoffs of P-value < 0.05 and an absolute value of $\log_2$(fold-change) ≥ 1 were used for differentially upregulated or downregulated genes for gene ontology analysis by subjecting the gene list to DAVID bioinformatics tool. For GSEA, the normalized FPKM lists of our and public gene sets (Molecular Signatures Database, MSigDB) were selected for downstream analysis.

Cell transplantation in the infarcted rat heart

Following protocols approved by Hualianke Biotechnology Animal Care and Use Care Committee (HLK-20201210-01), animal experiments were performed. All procedures involving animals were performed according to the NIH guidelines. The nude rats (NIH-Foxn1rnu) aged 6-8 weeks were purchased from Beijing Vital River Laboratory Animal Technology Co. (China). Three batches of animal models were assigned. The first batch of rats was anesthetized and thoracotomy injected EPSC-CM directly into the left ventricle,

and engraftment was assessed 4 weeks after cell transplantation (n = 6). Rats were anesthetized by 400 mg/kg Tribromoethanol intraperitoneal injection, intubated, and mechanically ventilated. 6-8×10^6 cells were mixed with a transplant cocktail (PBS and Matrigel (corning, 354262) for 1:1, containing 20 ng/mL VEGF (R&D, 293-VE), 20 ng/mL FGF2 (peprotech, 100-18B), 1 μM Y-27632 (Stem cell, 72308) and 1 μM CHIR-99021 (Tocris, 4423)) at a volume about 100 μL was injected into the left ventricle at two points. The chest was closed by sequentially suture of rib, muscle and skin with a 5-0 suture. Then rats were placed on heating pads to wake up. The following two batches of nude rats were assigned to both MI procedure and cell transplantation. We induced MI by permanently ligation of the left anterior descending artery (LAD) using a 7-0 polypropylene suture. Following Ligation of LAD, animals were randomly divided into three groups: MI + vehicle, MI + iPSC-CM, and MI + EPSC-CM, and cell suspensions or hydrogel controls were injected into the peri-infarct area at two points, respectively. Vehicle control was a 100 μL cocktail without cells. After LAD and cell injection, the cardiac function was evaluated by echocardiography before MI and 2 days after MI (short term) or 6 weeks after MI (long term) with VINNO V6 Vet system. After finishing the echocardiography, rats were euthanized for histological observation. For rat number in the short-term batch, MI + vehicle: n = 5; MI + iPSC-CM: n = 6; MI + EPSC-CM: n = 6. For rat number in the long-term batch, MI + vehicle: n = 6; MI + iPSC-CM: n = 5; MI + EPSC-CM: n = 6.

Histological analysis

For histological analysis, all heart samples were fixed in 4% PFA for 24-36h at 4°C, halved 1-2mm below the ligature knot, then dehydrated in ethanol and xylene series, and embedded in paraffin. Hearts were sectioned serially at a thickness of 5 μm, and 20 sections were collected at each location, then 100 μm intervals were discarded. At least three levels per heart were selected to assess infarction size. The level with largest graft area in each heart was used to evaluate engraftment area, apoptosis and mitochondrial morphology. Hematoxylin & Eosin staining and Masson staining were executed following standard procedures. In addition, immunostaining experiments were performed as described above. Graft cells was rigorously confirmed by dual-immunolabeling with a human-specific antibody against the nuclear marker KU80 (CST, 2180) and cardiomyocyte marker SAA (Sigma, A7811) or Cardiac troponin I (Abcam ab56357). Primary antibodies against ATPB (Abcam, ab14730), and TUNEL kit (Vazyme, A112-03) were used to detect mitochondrial and apoptosis in the graft, respectively. The infarcted area was calculated by using the circumference method with Masson staining images, as previously reported[4]. For engraftment area analysis, the KU80 and CTNT positive area was quantified, and the left ventricular cross-sectional area was calculated as the total area. To calculate the apoptosis rate of grafted cardiomyocytes, TUNEL positive cells within engraftments were analyzed. All immunostaining images were captured by confocal microscope (ZEISS LSM980), and analyzed by Fiji-ImageJ software.

Statistics

GraphPad Prism software (version 8.0.2) was used for statistical analysis. All data are expressed as mean ± SEM. Normality tests were performed for the continuous variables. Comparisons between two groups of continuous variables modeled by a normal

distribution were carried out by unpaired Student's *t*-test; multiple comparisons were assessed by the unpaired one-way ANOVA. Data that were not modeled by a normal distribution were carried out Mann-Whitney test, and the nonparametric Steel-Dwass test assessed multiple comparisons. *P* values of less than 0.05 were considered statistically significant.

References

1 Zheng, R. *et al.* Derivation of feeder-free human extended pluripotent stem cells. *Stem Cell Reports* **16**, 2410-2414 (2021).

2 Bian, W., Liau, B., Badie, N. & Bursac, N. Mesoscopic hydrogel molding to control the 3D geometry of bioartificial muscle tissues. *Nat Protoc* **4**, 1522-1534 (2009).

3 Zhang, D. *et al.* Tissue-engineered cardiac patch for advanced functional maturation of human ESC-derived cardiomyocytes. *Biomaterials* **34**, 5813-5820 (2013).

4 Ong, S. G. *et al.* Microfluidic Single-Cell Analysis of Transplanted Human Induced Pluripotent Stem Cell-Derived Cardiomyocytes After Acute Myocardial Infarction. *Circulation* **132**, 762-771 (2015).
